# Diet-induced impulsivity: Effects of a high-fat and a high-sugar diet on impulsive choice in rats

**DOI:** 10.1371/journal.pone.0180510

**Published:** 2017-06-29

**Authors:** Catherine C. Steele, Jesseca R. A. Pirkle, Kimberly Kirkpatrick

**Affiliations:** Department of Psychological Sciences, Kansas State University, Manhattan, Kansas, United States of America; The University of Tokyo, JAPAN

## Abstract

Impulsive choice is a common charactertistic among individuals with gambling problems, obesity, and substance abuse issues. Impulsive choice has been classified as a trans-disease process, and understanding the etiology of trait impulsivity could help to understand how diseases and disorders related to impulsive choice are manifested. The Western diet is a possible catalyst of impulsive choice as individuals who are obese and who eat diets high in fat and sugar are typically more impulsive. However, such correlational evidence is unable to discern the direction and causal nature of the relationship. The present study sought to determine how diet may directly contribute to impulsive choice. After 8 weeks of dietary exposure (high-fat, high-sugar, chow), the rats were tested on an impulsive choice task, which presented choices between a smaller-sooner reward (SS) and a larger-later reward (LL). Then, the rats were transferred to a chow diet and retested on the impulsive choice task. The high-sugar and high-fat groups made significantly more impulsive choices than the chow group. Both groups became more self-controlled when they were off the diet, but there were some residual effects of the diet on choice behavior. These results suggest that diet, specifically one high in processed fat or sugar, induces impulsive choice. This diet-induced impulsivity could be a precursor to other disorders that are characterized by impulsivity, such as diet-induced obesity, and could offer potential understanding of the trans-disease nature of impulsive choice.

## Introduction

Impulsive choice is a trans-disease process [1] that is a common characteristic of individuals who suffer from substance abuse [2–4], obesity [5–8], and gambling issues [3, 9–12]. Impulsive choice behavior is the propensity to choose a smaller-sooner (SS) reward even if choosing the larger-later (LL) reward is the more optimal choice, and it is thought that this pattern of behavior is a potential precursor to disease [1]. Impulsive choice is proposed to emerge from high delay discounting rates, with delay discounting referring to the loss of subjective reward value as a function of delay [13]. Over the past decade, there has been emerging evidence that people who are obese make more impulsive choices [5–8]. While it is clear that there is a relationship between obesity and impulsive choice, the direction and nature of the relationship is not clear. It is possible that 1) trait impulsive choice causes obesity, 2) obesity causes trait impulsive choice, or 3) some third factor, such as diet, causes both.

The primary variables implicated in the development of obesity are genetics, diet, and physical activity. Genetics are an unlikely explanation for the rapid increase of the obesity epidemic as genes have not changed substantially in recent decades. Thus, efforts have focused on environmental factors, such as calorie intake, diet composition, and physical activity, as these factors have changed substantially over the past three decades with changes in modern diets and lifestyle factors [14, 15]. Of these factors, diet is considered a primary driver of the obesity epidemic [16, 17] because the Western diet consumed by a majority of Americans consists of foods high in processed fats and sugars. Indeed, recent research in humans suggests that consumption of a diet high in fat and sugar is associated with impulsive choice behavior [18], thus supplying one potential explanation for the relationship between impulsive choice and obesity.

Unfortunately, many of the effects of diet are difficult to study in humans as dietary history cannot be easily controlled, especially long-term dietary exposure. In addition, human research is often correlational in nature, so an advantage of animal models (e.g. rodents) is to allow determination of disease etiology. Diet-induced animal models of obesity have been used in a wide range of studies to understand how diet affects behavior; these animal models can be used to manipulate dietary experiences and document causal effects of chronic dietary exposure on the brain, behavior, and physiology [19, 20].

To our knowledge, only one previous study has investigated the direct effects of diet on impulsive choice using a rat model [21]. Rats were either given a normal chow or high-fat diet for 8 weeks and then tested on an impulsive choice task. Their results indicated that some rats fed a high-fat diet gained more weight (obesity prone rats) and exhibited fewer impulsive choices than the obesity resistant rats and the normal chow group. This is contrary to the evidence in humans which shows that measures of obesity (i.e., BMI and body fat percentage) are associated with greater impulsive choice behavior [6–8]. Given the contradictory nature of these results, the present study sought to better understand this relationship between diet and impulsive choice by addressing some limitations in the previous study [21]. Our ultimate goal was to extend upon the well-established diet-induced obesity rodent models to develop a novel diet-induced impulsive choice model that can potentially be linked with the established human literature on diet, obesity, and impulsive choice as a trans-disease process.

## Methods

### Animals

Twenty-four male Sprague Dawley rats (Charles River, Kingston, NY) arrived at Kansas State University on post-natal day (PND) 28. The rats were housed individually in a dimly-lit (red light) colony room that was set to a reverse 12-hr light:dark schedule (lights off at approximately 7 am) with free access to water at all times. The rats were fed 25 g of rat chow daily from PND 28 to PND 127 during which time they were held in the colony room. Body weight was measured at least five times a week.

### Ethics statement

This study was carried out in accordance with the Guide for the Care and Use of Laboratory Animals of the National Research Council. The protocol was approved by the Kansas State University Institutional Animal Care and Use Committee (Protocol 3490).

### Experimental timeline

**Fig 1:**
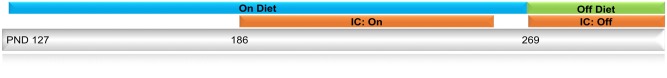
Experiment timeline. Rats received initial dietary exposure for 8-weeks (PND 127–182) before beginning the impulsive choice task on the diet (IC: On; PND 186). Following removal from the diet and transition to an all chow diet (PND 268), rats completed the impulsive choice task off the diet (IC: Off; PND 269).

Fig 1 depicts the timeline of the experiment. Rats received the initial dietary exposure for 8 weeks (PND 127–182). Following dietary exposure, rats completed pre-training (PND 183) before beginning the impulsive choice (IC) task on the diet (PND 186). The rats were then removed from the diet (PND 268) and began the impulsive choice (IC) task off the diet beginning on PND 269.

### Dietary conditions

Rats were randomly assigned to one of three groups (*n* = 8) to determine the separate effect of fat and sugar on behavior: High-Fat (HF), High-Sugar (HS) and Chow (C). To determine the number of subjects required for each group to obtain statistically significant results at the p < .05 level (assuming effect sizes of .5-.7 and observed power of > .8), we conducted analyses of effect size and power on samples of previous data from Jurdak, Lichtenstein, & Kanarek (2008), whose dietary manipulations were similar to the current study. Based on our analyses, we required a minimum of 8 rats per condition to ensure that we were able to obtain robust group differences combined with the ability to observe a range of variation in the performance of individual rats. Body weight and general locomotor activity (assessed in a locomotor chamber) were checked to ensure statistically similar groups prior to dietary exposure (data on locomotor activity not shown).

#### Initial dietary exposure

All rats received access to 101.75 calories each day to minimize differences in energy budget. Group C received 25 g of standard rat chow (4.07 kcal/g), Group HS received 15 g of chow and 10.33 g of sucrose (3.94 kcal/g) mixed into unflavored gelatin, and Group HF received 15 g of chow and 4.38 g of hydrogenated vegetable fat (9.3 kcal/g) [20]. The chow was given in the food hopper, and the supplements (hydrogenated vegetable fat or sucrose mixed into unflavored gelatin) were given in plastic dishes placed on the floor of the cage. Group C received 10 g of their chow in a plastic dish placed on the floor of the cage to control for the experience with the bowls. The rats received their designated diet for 8 weeks in the home cage from PND 127 to PND 182 (see Fig 1). Four weeks before behavioral testing, food access was restricted so that the rats were allowed 4 hr of access during the first week of restriction, 3 hr in the second week, and 2 hr during the two weeks prior to behavioral testing. The goal was to control for the time between dietary consumption and the subsequent behavioral testing session so that all rats completed consumption of their diets approximately 20 hr before the start of the next session. By controlling access, we aimed to minimize any acute effects of the recent diet consumption on motivational processes.

#### Dietary exposure during behavioral testing

The rats completed the impulsive choice task while maintained on the dietary manipulation to assess the chronic effects of the diet exposure (Fig 1). During behavioral testing, rats earned 45-mg grain-based pellets (F0165, Bio-Serv, Flemington, NJ) in the operant chambers. The number of grams earned in the chambers was subtracted from their daily chow ration, such that the total amount of food received in the chambers and on the food hopper in the home cages was 15 g. Following behavioral testing each day, the rats received the remainder of their chow plus their dietary supplement in the home cages.

After the completion of the initial impulsive choice task, all rats were removed from the dietary manipulation and fed an all chow diet for one day (Fig 1). The rats then completed the choice task again while on an all chow diet to assess whether the dietary exposure effects lingered after the diet was removed. The rats continued to receive 15 g of chow each day between food earned in the experiment plus supplement feeding in the food hopper of the home cage. The additional 10 g of chow was placed in a bowl on the cage floor as a supplement to match the food exposure in the on diet phase.

#### Food consumption

Bedding was scanned for spillage of the supplements each day, and any spillage was removed and noted. However, because spillage of the supplements was often intermixed with bedding, we were unable to accurately quantify the spillage. Generally, Group C reliably ate all of their 10 g of chow from the bowl and Group HF ate all of their fat supplement. Group HS typically consumed the majority of their sugar supplement, but often left some supplement uneaten.

Any leftover chow in the food hopper was weighed and recorded to determine food intake. Chow intake during the final week of initial dietary exposure was analyzed to determine if the groups differed in chow consumption prior to the onset of behavioral testing. There were no significant differences in chow intake between Group C (*M* = 7.125, SE = .392), Group HF (*M* = 7.839, SE = .392), and Group HS (*M* = 8.102, SE = .419), *F*(2,160) = 1.588, *p* = .208. Chow consumption was not analyzed during behavioral testing because rats earned a considerable portion of their daily chow ration in the experiment, thus complicating the interpretation of chow consumption in the home cage.

### Apparatus

The impulsive choice task was conducted in 24 operant chambers (Med-Associates, St. Albans, VT), each housed within a sound-attenuating, ventilated box (74 × 38 × 60 cm). Each operant chamber (25 × 30 × 30) was equipped with a stainless steel grid floor; two stainless steel walls (front and back); and a transparent polycarbonate side wall, ceiling, and door. Two pellet dispensers (ENV-203), mounted on the outside of the front wall of the operant chamber, delivered 45-mg food pellets to a food cup (ENV-200R7) that was centered on the lower section of the front wall. Two retractable levers (ENV-112CM) were located on opposite sides of the food cup. The chamber was also equipped with a house light (ENV-215) that was centered at the top of the chamber’s front wall, as well as two nose-poke key lights (ENV-119M-1) that were each located above the left and right levers. Water was always available from a sipper tube that protruded through the back wall of the chamber. Experimental events were controlled and recorded with 2-ms resolution by the software program MED-PC IV [22].

### Procedure

#### Pre-training

Prior to the onset of the initial choice task during the dietary exposure, the rats were given preliminary training to collect pellets from the feeder and press the levers. During the first pre-training session, rats received magazine training and lever-press training. The magazine training consisted of delivery of 60 food pellets into a food cup through a random time 60-s schedule. Following magazine training, lever-press training rewarded lever presses with a fixed-ratio (FR) 1 schedule of reinforcement. Left and right levers were trained separately in six randomly alternating sub-blocks of 10 food deliveries resulting in a total of 30 pellets per lever. Following the first pre-training session, the rats continued lever-press training for two sessions. First, lever pressing was rewarded on an FR1 schedule of reinforcement. Left and right levers were trained separately in four sub-blocks (two blocks per lever, randomly alternating). Following the FR1 training, both levers were inserted and lever pressing was rewarded on a random ratio (RR) 3 schedule of reinforcement, followed by an RR5 schedule of reinforcement. For both the RR3 and RR5 block, each of the four sub-blocks consisted of 5 food deliveries per lever.

#### Impulsive choice (IC) task

The impulsive choice task was used to evaluate the rats’ preference for the SS versus LL rewards. The impulsive choice task was completed while maintained on the dietary manipulation (IC: On) and repeated again while the rats were maintained on an all chow diet (IC: Off). Each session consisted of a randomly intermixed series of free choice and forced choice trials. On free choice trials, both the left and right levers were inserted into the chamber, corresponding to SS and LL outcomes, with lever assignments counterbalanced across rats. Upon selection of one of the outcomes via a lever press, the other lever was retracted. The choice initiated a delay until food was available to be delivered. The first lever press following this delay caused the lever to retract, food to be delivered, and a 60-s intertrial interval (ITI) to begin. Forced choice trials were identical to free choice trials, except that only one lever was inserted into the chamber. Each session consisted of 54 free choice trials, 14 SS forced choice trials, and 14 LL forced choice trials, and lasted until all 82 trials had been completed or approximately 2 hr had elapsed. The SS reward was 1 pellet and the LL reward was 2 pellets. The LL delay was 30 s and the SS delay increased across phases: 5, 10, and 20 s.

Each phase lasted until rats achieved stable performance, where choices on the last three sessions were within ±10% of the mean of those sessions. While rats were on the dietary manipulation, Phases 1–3 lasted 15, 27, and 20 sessions, respectively. During the off diet testing, Phases 1–3 lasted 10, 15, and 10 sessions, respectively. The off diet testing was shorter because rats achieved stable performance quicker when off the diet.

### Data analysis

The raw data were imported into MATLAB R2016a (The MathWorks, Natick, MA), and generalized linear regression modeling was conducted on the body weight and impulsive choice data with a random intercept to account for individual differences. The impulsive choice analyses used a binomial distribution with a logit link function, and the body weight analyses used a normal distribution with a linear link function. Group and On/Off diet were coded as categorical and were effect-coded in all analyses (i.e., the coefficients summed to 0).

For the choice analyses, we adapted an approach developed by Wileyto et al. [23] for generalized linear modeling of delay discounting/impulsive choice functions. Their approach computes separate terms for delay sensitivity (slope of the function) and magnitude sensitivity rather than a single parameter (e.g., k-value, or discounting rate) to better isolate potential mechanisms of choice behavior. The slope of the function in the present study served as a measure of the delay discounting rate as well as a measure of delay sensitivity. Because we did not manipulate the magnitude of reward, instead of using a model term for magnitude sensitivity, we used the intercept as a constant to capture the predicted biases in choice at a 0-s SS delay, a measure of “bias for immediacy.” Accordingly, SS delay was coded as a continuous variable and was scaled as a proportion of the LL delay (SS/LL) so that the estimates for slope represent sensitivity to delay across the whole choice function and the estimates for the intercept represent predicted choice biases at an immediate (0-s) SS delay. For the body weight analyses, age was coded as a continuous variable and was mean-centered.

Because the variables were effect coded so that the dummy codes add to zero, an omnibus analysis was conducted to test for overall effects and then post-hoc tests comparing each of the diet groups with Group C were conducted using the coefficient test function in MATLAB. Any differences between Group HF and Group HS were not directly tested as we were primarily interested in the effects of the unhealthy diets relative to the control diet. The unstandardized coefficients (*b*-values) and the associated 95% confidence intervals are reported as the measure of effect size; this is the recommended measure of effect size for generalized linear models with a binomial distribution and a logit link [24]. Note that the *b*-values are log odds ratios so that negative values for the intercept and main effects reflect preference for the SS, positive values reflect preference for the LL, and zero is indifference.

For all figures, the error bars surrounding the means were computed using the Predict function in MATLAB which provides the standard error (SE) of the estimates from the generalized linear model. Thus, the SE values represent the confidence for the model predictions for each mean in the data set. We only report test statistics for variables that were significant at the p < .05 level.

The choice analysis included all choices made during the last five sessions of each SS delay. We examined choice behavior both within and across sessions to see if the diets may have affected choice dynamics. We observed that the rats showed learning of the choice parameters within and across the early sessions of each SS delay and then stabilized by the end of each SS delay phase (both within and across sessions). There were no appreciable effects of the diet on acquisition of choice behavior so we focused the analysis on the asymptotic choice behavior to capture the predominant patterns in the data. We analyzed body weights across age as there were some differences in the rate of weight gain during the dietary phase.

One rat from Group HS was removed from all analyses. His choice behavior was highly biased and unsystematic, and he did not reliably consume the sugar dietary supplement. It was subsequently discovered when perfusing the brains for a separate pilot study that there were severe neuronal abnormalities in his brain (grossly enlarged ventricles), which most likely explained his aberrant choice behavior.

## Results

### Impulsive choice

#### On versus off diet

**Fig 2:**
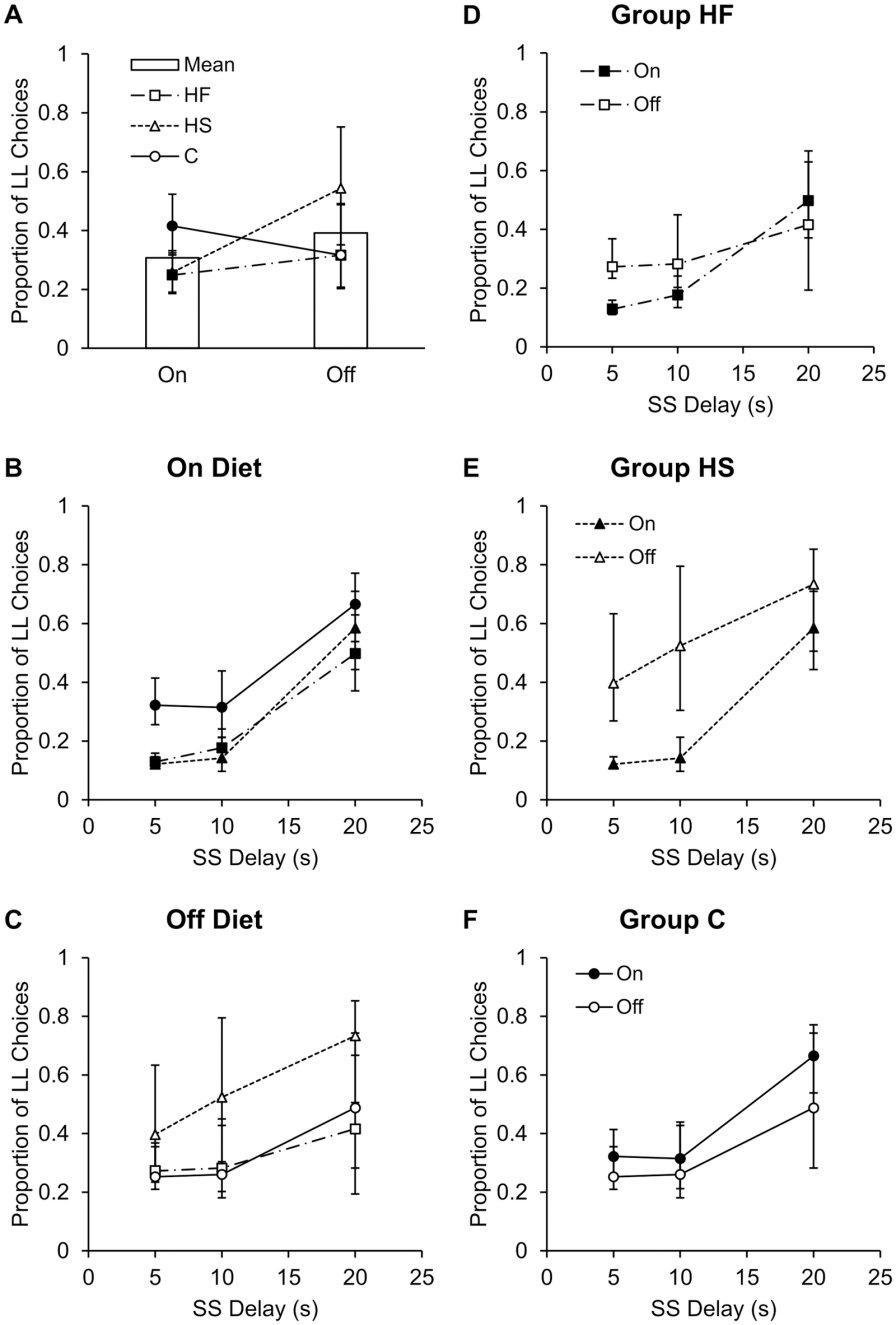
Dietary effects on impulsive choice. A) Mean proportion of LL choices overall and for each group when rats were tested on their designated diets versus when tested off the diets. HF and HS rats became less SS preferring after removal from the diet compared to C rats (*p*s < .001). B) Mean proportion of LL choices as a function of SS delay for each group when rats were tested on their designated diets. HF and HS rats made more impulsive choices, indicating a greater bias for immediacy than C rats (*p*s = .021 and .003, respectively). HF and HS rats also had steeper slopes than C rats (*p*s < .001), indicating heightened sensitivity to delay. C) Mean proportion of LL choices as a function of SS delay for each group when rats were tested off their designated diets. There were no significant differences in bias, but HF and HS rats had steeper slopes than C rats (*p* = .016 and *p* < .001, respectively). D) Mean proportion of LL choices for Group HF as a function of SS delay when they were on versus off the diet. HF rats made fewer impulsive choices and displayed lower slopes off the diet (*p*s < .001). E) Mean proportion of LL choices for Group HS as a function of SS delay when they were on versus off the diet. HS rats made fewer impulsive choices and displayed lower slopes off the diet (*p*s < .001). F) Mean proportion of LL choices for Group C as a function of SS delay when they were on versus off the diet. C rats showed no differences in impulsive choice on and off the diet (*p* = .761), but they had lower slopes off the diet (*p* < .001). Errors bars depict ± the SE of the model prediction.

Fig 2A displays the effect of the dietary manipulation on the overall proportion of LL choices (data in S1 Text). Overall, the rats made more LL choices when they were off the diet than when they were on the diet, *t*(92721) = 41.12, *p* < .001, *b* = 0.92 [0.88, 0.97]. However, the changes in the groups were not uniform, as evidenced by a significant Group × On/Off interaction. Pairwise coefficient tests indicated that Group HF, *F*(1,92721) = 531.17, *p* < .001, and Group HS, *F*(1,92721) = 904.03, *p* < .001, both differed in the on versus off diet phases compared to Group C. When off diet, Group HF became less SS preferring overall (on: *b* = -4.88; off: *b* = -2.40) so that they were now similar to Group C (on: *b* = -2.37; off: *b* = -2.39). Group HS also became less SS preferring overall, and they were now less SS preferring than Groups HF and C (on: *b* = -4.66, off: *b* = -1.57; see Fig 2A). In addition, overall sensitivity to delay was lower in the off diet phase compared to the on diet phase, On/Off × SS Delay, *t*(92721) = -26.85, *p* < .001, *b* = -1.38 [-1.48, -1.28]. There also was a Group × SS Delay interaction and a Group × On/Off × SS Delay interaction; the three-way interaction is shown in Fig 2B and 2C. Additional models were conducted to explore group differences.

#### On diet

Fig 2B shows the proportion of LL choices as a function of SS delay for all groups during the on diet phase. There was a significant main effect of group, indicating differences in choice biases (intercept) during the on diet phase. Coefficient tests indicated that Group HF, *b* = -3.99, made more impulsive choices compared to Group C, *b* = -2.25, *t*(57249) = 2.31, *p* = .021, as did Group HS, *b* = -4.56, *t*(57249) = 2.96, *p* = .003. There was also a significant Group × SS Delay interaction which indicates differences in sensitivity to delay (i.e., delay discounting rate), and coefficient tests indicated that Group HF displayed a significantly steeper slope (greater sensitivity to delay/steeper discounting), *b* = 5.75, compared to Group C, *b* = 4.41, *t*(57249) = 8.43, *p* < .001, as did Group HS, *b* = 7.48, *t*(57249) = 18.55, *p* < .001. Overall, Groups HF and HS showed greater sensitivity to delay/steeper discounting when on the diet, and Groups HF and HS made fewer LL choices compared to Group C.

#### Off diet

Fig 2C displays the proportion of LL choices as a function of SS delay for all groups during the off diet phase. There was no significant main effect of group, indicating that the Groups HF, *b* = -3.56, and HS, *b* = -2.28, did not differ from Group C, *b* = -3.35, in their bias for immediacy (intercept at 0). There was a significant Group × SS Delay interaction, and coefficient tests indicated that Group HF displayed a steeper slope, *b* = 4.95, indicating greater sensitivity to SS delay/steeper discounting compared to Group C, *b* = 4.33, *t*(35472) = 2.42, *p* = .016, as did Group HS, *b* = 5.58, *t*(35472) = 5.29, *p* < .001. Overall, Groups HF and HS continued to show enhanced sensitivity to delay off the diet compared to Group C. However, the bias for immediacy did not differ between the groups off the diet.

#### On versus off diet by group

To better understand the diet effects within each group, we also conducted generalized linear models for each group with the variables of on/off and SS delay. Fig 2D–2F displays the comparisons of on versus off diet for each group. Group HF made significantly more LL choices, *t*(33271) = 28.60, *p* < .001, *b* = 1.24 [1.15, 1.32] and displayed lower slopes (i.e., sensitivity to delay/discounting rate), *t*(33271) = -22.58, *p* < .001, on: *b* = 7.17, off: *b* = 2.94, in the off diet phase (see Fig 2D). Group HS also made significantly more LL choices, *t*(33271) = 38.43, *p* < .001, *b* = 1.54 [1.47, 1.62] and displayed lower slopes, *t*(33271) = -13.87, *p* < .001, on: *b* = 7.30, off: *b* = 4.70, in the off diet phase (see Fig 2E). Finally, for Group C, there was no significant difference in impulsive bias on versus off diet, *t*(33271) = -0.30, *p* = .761, *b* = -0.01 [-0.07, 0.05], but there were lower slopes during the off diet phase, *t*(33271) = -9.14, *p* < .001, on: *b* = 4.54, off: *b* = 3.11 (see Fig 2F). Overall, all three groups showed lower slopes (sensitivity to delay/delay discounting) during the off diet phase, and Groups HF and HS showed higher intercepts (i.e., an increased LL choices) when they were off the diet.

### Body weight

#### On versus off diet

**Fig 3:**
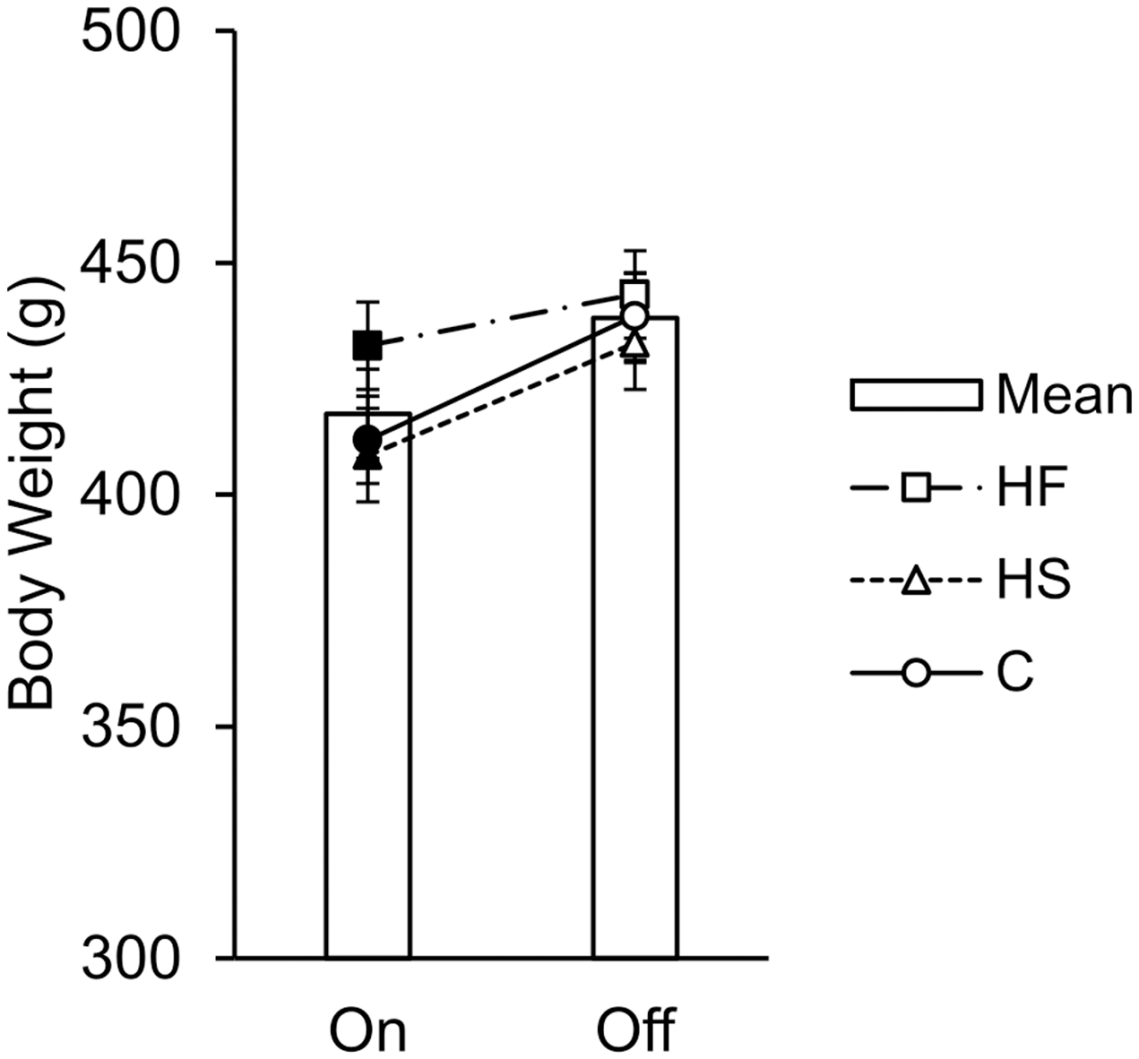
Dietary effects on overall body weight on and off the diet. Mean body weight (± the SE of the model prediction) while the rats were maintained on their designated diets versus when maintained off the diets. All groups weighed more off the diet (*p* < .001). The change in weight from on to off for the HF rats was smaller than C rats (*p* < .001), but the degree of change in weight for HS rats did not differ from C rats (*p* = .973).

**Fig 4:**
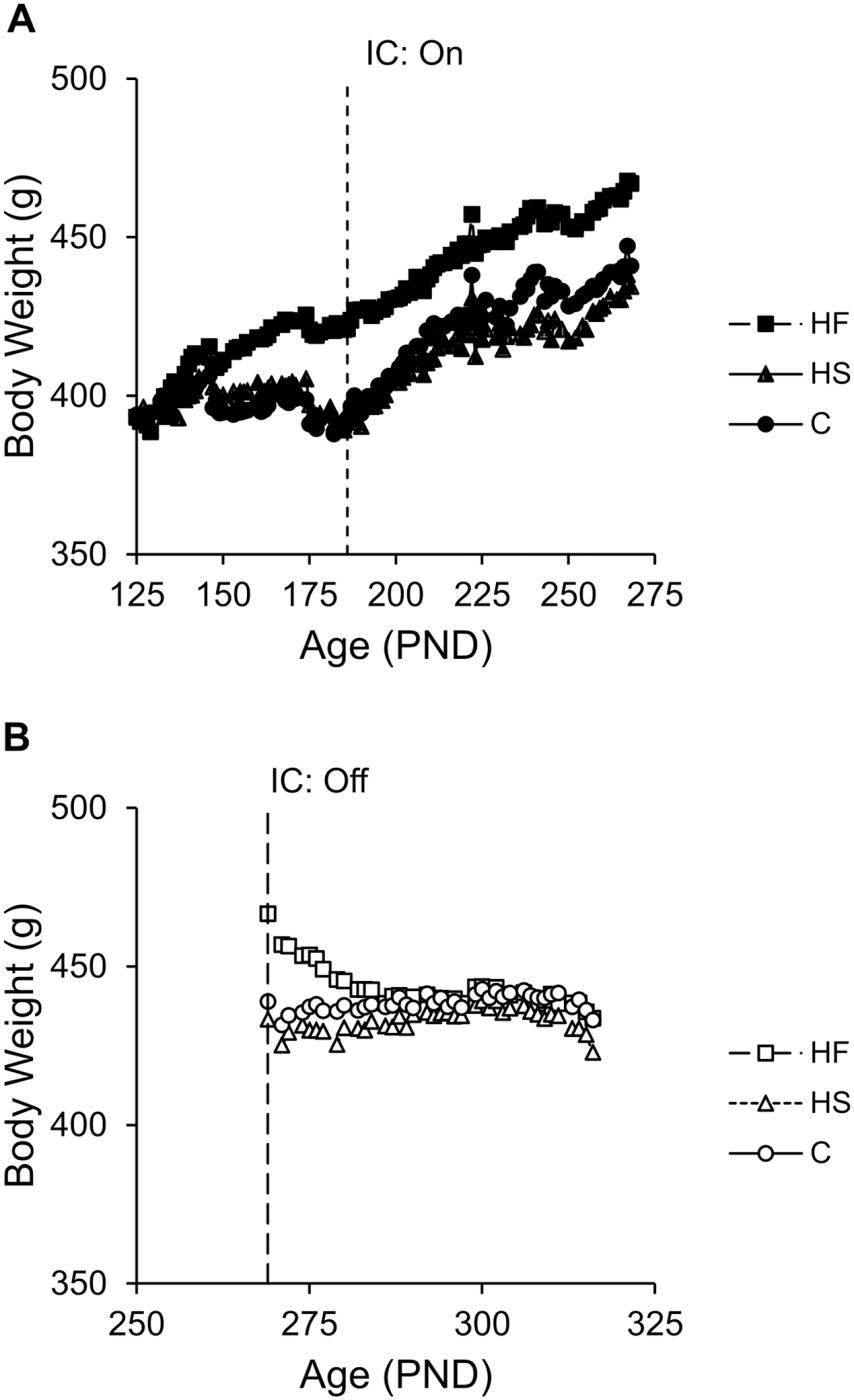
Dietary effects on body weight as a function of age. A) Mean body weight for each group as a function of age when rats were on the diets. HF rats gained weight more quickly than C rats (*p* < .001), and C rats gained weight more quickly than HS rats (*p* < .001). B) Mean body weight for each group as a function of age when rats were off the diets. HF rats showed a decrease in weight across the off diet phase compared to C rats (*p* < .001), and HS rats did not differ from C rats (*p* = .969). The beginning of the impulsive choice (IC) task during the on and off diet phases is depicted by the vertical, dashed line.

The analysis revealed a main effect of on/off (see mean data in Fig 3), such that all groups weighed more during the off diet phase than during the on diet phase, *t*(3484) = 9.58, *p* < .001, *b* = 8.09 [6.43, 9.75]. The increase in body weights from the on diet phase to the off diet phase was most likely due to the effect of age, *t*(3484) = 13.02, *p* < .001, *b* = 0.15 [0.13, 0.17], as the rats steadily gained weight as they were growing into early adulthood during the on diet phase (Fig 4A). Note that there was a reduction in body weight across all groups prior to behavioral testing, likely a result of the restricted feeding access time to ensure that the rats were motivated to perform in the choice task (Fig 4A). There also was a Group × On/Off interaction. Pairwise coefficient tests indicated that the difference in body weight between on and off diet was significantly smaller in Group HF compared to Group C, *t*(3484) = 3.73, *p* < .001, but that Group HS did not differ from Group C, *t*(3484) = 0.03, *p* = .973. Finally, there was a three-way interaction between group, on/off, and age (data in S2 Text). Subsequently, body weights on and off the diet were modeled separately to follow up the 3-way interaction.

#### On diet

There was an overall effect of age, *t*(2593) = 85.47, *p* < .001, *b* = 0.37 [0.36, 0.38] and a Group × Age interaction. The interaction is displayed in Fig 4A. Coefficient tests on the interaction confirmed that Group HF, *b* = 0.48, gained weight at a faster rate than Group C, *b* = 0.36, *t*(2593) = 11.17, *p* < .001, and Group C gained at a faster rate than Group HS, *b* = .26, *t*(2593) = 9.98, *p* < .001.

#### Off diet

During the off diet phase, body weights declined as a function of age, *t*(891) = -4.93, *p* < .001, *b* = -0.07 [-0.09, -0.04]. The diet groups responded differently to the removal of their dietary supplements. Specifically, as seen in Fig 4B, body weight decreased during the off diet phase in Group HF but increased gradually in the other two groups. Accordingly, there was a significant Group × Age interaction, and pairwise comparisons confirmed that Group HF, *b* = -0.38, displayed a negative slope compared to Group C, *b* = 0.09, *t*(891) = 14.77, *p* < .001. Group HS, *b* = 0.09, did not differ from Group C, *t*(891) = 0.04, *p* = .969.

### Individual differences

**Fig 5:**
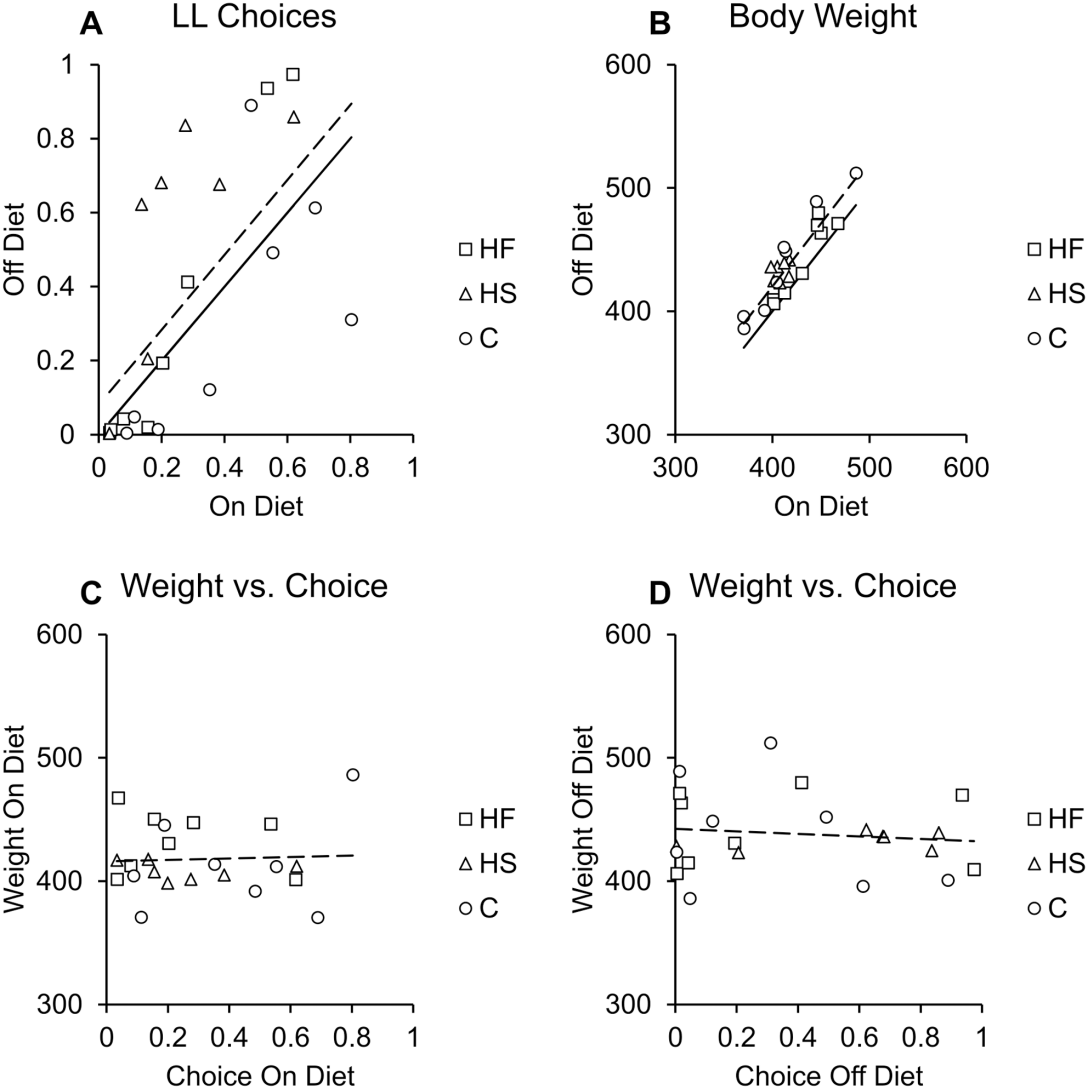
Individual differences in body weight and impulsive choice. A) The correlation between the proportions of LL choices made on and off the diet (*p* = .001). B) The correlation between body weights on and off the diet (*p* < .001). C) The correlation between body weight and choice on the diet (*p* = .834). D) The correlation between body weight and choice off the diet (*p* = .601).

There were considerable individual differences in choice behavior and body weight as evidenced by the inclusion of a random intercept to the models. The individual differences were stable across the on and off diet manipulations. The stability of individual differences was evidenced by a significant correlation between choice behavior on and off the diet, *r* = .66, *p* = .001 (Fig 5A) and a significant correlation between weight on and off the diet, *r* = .92, *p* < .001 (Fig 5B). However, there was no relationship between weight and choice during the on diet phase, *r* = .05, *p* = .834, or during the off diet phase, *r* = -.12, *p* = .601 (Fig 5C and 5D).

## Discussion

With many Americans consuming Western diets high in processed fat and sugar, the current study sought to understand how diet affects impulsive choice behavior to potentially elucidate the relationship between obesity and impulsive choice that has been found in humans [5–8]. This is critical as self-control is an important variable in many health behaviors including diet and exercise [25] and impulsive choice has been implicated as a trans-disease process [1]. In addition, we were able to parse out the effects of diet on bias versus sensitivity to delay (delay discounting rates) to provide an insight into potential mechanisms of diet effects on choice.

With regard to impulsive choice bias, Groups HF and HS rats were more impulsive when on the diet than rats fed a normal chow diet (Fig 2A). However, when the HF and HS groups were placed on a normal chow diet their bias no longer differed from the control diet (Fig 2A), suggesting that impulsive bias was under strict control of the dietary conditions. The measure of impulsive choice bias used in the present study was the predicted LL choices at a 0-s delay (i.e., the intercept of the regression model), providing an index of bias for immediate rewards. Because the ITI was fixed at 60 s, choosing the LL would result in greater overall reward earning in all cases even at a 0-s delay where the choice would be between 1 pellet every 60 s versus 2 pellets every 90 s. Thus, the HF and HS diets produced behavior that was more sub-optimal compared to the control diet. Overall, the pattern of results indicates that the HF and HS diets induced a bias for immediate rewards, but this effect did not persist when the diet was removed. Interestingly, Group HS showed an impulsive bias in the absence of any increases in body weight compared to Group C, and indeed Group HF only gained modest body weight. This indicates that the effects of the diet on self-control were separable from the effects on body weight.

Groups HF and HS also showed enhanced sensitivity to delay in that they had a steeper overall slope compared to Group C (Fig 2B) during the on diet phase, and both groups continued to show greater sensitivity to delay during the off-diet phase compared to Group C (Fig 2C). Enhanced sensitivity to delay is an indicator of steeper discounting of delayed rewards [13], a hallmark of impulsive choice. Steeper discounting functions are associated with obesity in humans [5–8]. The present result provides some explanation of these correlational patterns by demonstrating that the HF and HS diets increased delay sensitivity and caused an impulsive bias. Thus, this diet-induced impulsivity model supplies an important potential explanation for the relationship between impulsive choice and obesity observed in humans. Although impulsive bias was normalized in the HF and HS groups, the continued elevation of delay sensitivity suggests a persistent effect of the diets on choice behavior in increasing the discounting rate through increasing delay sensitivity. Thus, the diet effects on choice were only normalized in the impulsive bias measure, but not in the delay sensitivity/delay discounting measure. Further work is needed to determine the specific mechanisms of the short- and long-term dietary effects on choice, but there is growing evidence of a relationship between delay sensitivity and impulsive choice [26–29] so timing processes may be a suitable target for further investigation.

Another possible driver of at least some aspects of the choice behavior in Group HS may be the sugar content in the test pellets; 30% of the total energy in the pellets was from mono- or di-saccharides, so the sugar content in the pellets may have been especially attractive to Group HS. However, one would then expect that Group HS should have made more LL choices when on the diet if they were seeking the sugar in the pellets, as LL choices are more optimal in terms of reward-earning and they should have preferred to receive the 2-pellet LL reward over the 1-pellet SS reward. It is possible, however, that the off diet results in Group HS may have been affected by sugar-seeking. Specifically, Group HS made more self-controlled choices following removal from the diet compared to the other two groups (Fig 2A). The sugar content in the pellets may have been reinforcing because they were no longer receiving a sugar supplement, thus resulting in increased desirability of the sugar content in the test pellets [30] and as a result increased LL choices. It is worth noting, however, that Group HS still showed enhanced sensitivity to delay similar to their on diet phase, so any preference for the sugar within the test pellets is not likely the source of those results. Morever, the sugar content of the pellets does not provide any convincing explanation for the results of Group HF. Thus, this effect may be limited solely to the increased LL bias observed in Group HS after removal from the diet, but does provide an interesting potential account of that finding.

In addition to the effects of diet on choice, all three groups showed reduced sensitivity to delay during the off diet phase (Fig 2C–2F). The decrease in delay sensitivity when off the diet may be a result of long-term effects of single housing which can lead to shallower impulsive choice functions compared to social housing conditions [31, 32]. Alternatively, the effects may be due to re-testing of choice behavior or due to additional learning of the choice options. In any event, these effects are likely not the source of the increases in self-control in the HF and HS groups during the off diet phase because all three groups were given equivalent exposure to these factors.

The body weight results indicated that rats fed a high-fat diet gained significantly more weight than rats fed a high-sugar or normal chow diet during the on diet phase (Fig 3). However, after removal from their high-fat diet, body weight normalized (Fig 3). While the rats fed a high-fat diet gained significantly more weight as they aged, thus showing signs of a trajectory towards obesity, their individual body weights did not correlate with impulsive choice. The test-retest correlations for choice and body weight indicate that the measurements were reliable, thus showing that the diets did not affect the expression of individual differences within measures. In humans, measures of obesity, such as BMI, often correlate with impulsive choice behavior. The lack of correlation between choice and body weight may be because the dietary exposure did not induce obesity. Due to a desire to control for calorie intake and energy budget, which has been shown to affect choice behavior [33], the rats had limited exposure to the supplements and thus all of the groups gained limited weight. Even the high-fat group, which gained the most weight, were still far from obese. The fact that the diets impacted on choice behavior indicates that the behavioral (neurological) effects of the diet were apparent well before any significant effects of the diet on body weight, and may possibly be a harbinger of a pathway towards obesity in that diet-induced impulsivity may be a precursor to diet-induced obesity or other dieases charactertized by impulsivity.

While body weight is typically used in diet-induced models of obesity as the measure of obesity in rats, it may not be the best measure [34]. The effects of diet on body weight are inconsistent such that some studies suggest that a high-fat and a high-sugar diet will result in weight gain while others suggest that only a high-fat diet will lead to weight gain [20, 35], as was observed in the present study. Body composition (e.g., body fat percentage) may be a more appropriate measure, as body composition can differ without seeing major differences in weight. This may explain why Group HS did not gain more weight than Group C as their body composition may have been altered even though their body weight was not affected by the diet. It has been proposed that percent body fat is under greater dietary control than body weight, and it may be a better measure of obesity in rats [36]. Similarly, in human research, there is a robust correlation between body fat percentage and impulsive choice, whereas there is not always a correlation between BMI and impulsive choice [6, 37]. Thus, it would be valuable to measure body fat percentage under the exposure to the present diets to see if the diet exposure may alter body composition even though there are only modest effects on body weight.

It is clear that high-fat and high-sugar diets induce impulsive behavior. This suggests that the relationship between obesity and impulsive choice may result, at least in part, from consumption of high-fat and high-sugar foods. Future research should investigate the effects of the Western diet (combined high-fat/high-sugar) on impulsive choice. Diet-induced impulsivity could be a pathway to obesity and other diseases associated with impulsive choice. Future studies should investigate the effect of diet-induced impulsivity on other diseases with impulsive choice as a trans-disease process as well as understanding the pathway from diet-induced impulsivity to obesity.

## Supporting information

S1 Text Impulsive choice data. (XLSX)

S2 Text Body weight data. (XLSX)
